# Health promoting schools and COVID-19: preparing for the future

**DOI:** 10.1177/14034948221091155

**Published:** 2022-05-01

**Authors:** Marjorita Sormunen, Lynda Lattke, Karina Leksy, Kevin Dadaczynski, Evanthia Sakellari, Veronica Velasco, Katarzyna Borzucka-sitkiewicz, María J. Miranda-velasco, Emanuela Rabaglietti, Rafaela Rosário

**Affiliations:** 1 Institute of Public Health and Clinical Nutrition, University of Eastern Finland, Kuopio, Finland; 2 Department of Psychology, University of Turin, Turin, Italy; 3 Institute of Pedagogy, University of Silesia, Katowice, Poland; 4 Department of Health Science, Fulda University of Applied Sciences, Fulda, Germany, and Centre for Applied Health Science, Lueneburg, Germany; 5 Centre for Applied Health Science, Leuphana University Lueneburg, Lueneburg, Germany; 6 Department of Public and Community Health, University of West Attica, Athens, Greece; 7 Department of Psychology, Milano-Bicocca University, Milan, Italy; 8 Department of Educational Sciences, University of Extremadura, Extremadura, Spain; 9 School of Nursing, University of Minho, Minho, Portugal; 10 Health Sciences Research Unit: Nursing (UICISA: E), Nursing School of Coimbra (ESEnfC), Coimbra, Portugal

**Keywords:** Health promoting schools, COVID-19, pandemic, children, family, teacher, school leader, non-teaching staff, school community

## Abstract

We face an unprecedented period of history during which COVID-19 is clustered with other global conditions, such as obesity, undernutrition, an infodemic, and climate change. This syndemic (synergy of epidemics) calls for the development of children’s and youth’s health literacy and socioemotional skills, support for behavioural hygiene (e.g. washing hands, wearing masks), and adults’ responsibility and caring. Moreover, it calls for creating conditions for healthy living and learning for all and paying extra attention to inequalities that have increased during the pandemic. Today, more than ever, there is an essential demand for schools to create environments that maintain and promote health for all. Within this commentary, we argue that whole-school approaches, such as the health promoting school, are essential to fight against the pandemic and to prepare schools for future challenges.

## Introduction

In 2020, the novel COVID-19 spread rapidly worldwide. In the first half of 2022, countries are still in different stages of the pandemic. Even though the vaccinations are being distributed, new variants are emerging and causing insecurity and restrictions [1, 2]. With its many physical, psychological, social, and economic impacts, the pandemic has detrimentally affected the lives of people. In particular, it has threatened the wellbeing of many children and youth in numerous unprecedented ways. As children and youth normally spend a substantial amount of time at school, partial or full school closures around the globe [3, 4] may have contributed to a learning loss in children’s education, in turn widening the inequality gap [5, 6].

Much has been done in recent decades to create a supportive school environment for healthier growth and learning [7]. Along with educational objectives, the unique role of schools has been widely acknowledged as being an ideal arena for the promotion of health among children and youth, as well as among school staff and the wider community [8, 9]. We acknowledge the whole-school approach to health, which holds that all components of the school community can contribute greatly to health and wellbeing and agree on the recently published recommendations that the principles of health promoting schools (HPSs) are even more important during a pandemic [10]. In this commentary, we explore the perspectives of different groups involved in and with schools (i.e. children and youth, their parents and caregivers, schools’ teaching and non-teaching staff, and school leaders) in relation to COVID-19.

## Families in the midst of pandemic

COVID-19 has drastically affected families. Services and activities that are usually provided to families at the national, regional, or local level, such as work and education, have been either fully or partially addressed at home. These structures people relied on and around which they organised their daily lives have either changed or become inactive [11], leading to an increase in stressors inside the family [12], and creating insecurity about the future. In addition to children’s schooling, most other school events and activities have been moved online and considered the ‘new normal’, even though digital platforms cannot fully replace face-to-face communication [11]. The remote education may have compromised children’s learning with potential negative effects for parents’ stress and complaints [13–15].

The pandemic and related infodemic, that is, the overwhelming amount of information about COVID-19 circulating online [16], are particularly acute for children and youth, who may not understand the orders and bans imposed on society, such as the closure of schools and kindergartens. Moreover, children are susceptible to the disordered information ecosphere, including disinformation, misinformation, and rumours. National lockdowns have been particularly disadvantageous for children’s wellbeing and psychological development [17, 18]. This situation has had many adverse consequences related to the effectiveness of learning and the mental health of children and youth [19]. Most notably, emotional support from family members, teachers, other adults, and significant peers might have been inadequate due to home confinement, and daily routines may have been missing.

In addition, children’s lifestyles may have changed [20], along with increasing worries, helplessness, fear and anxiety, especially among vulnerable individuals [18, 21]. The absence of the interactions that take place at schools may have led to decreased social support, resulting in increased social isolation and even violence at home. This sequence has been confirmed by more cases of physical, mental, and sexual violence against children during the pandemic [19]. Physical and social isolation may also have affected the cognitive development of children and adolescents [22].

Parents/caregivers are an integral part of the school community. Parental involvement has typically involved activities at home and at school aimed at supporting children’s educational development. However, during the school closures, which forced children to stay at home, not all parents were able to supervise their children at home. Moreover, in terms of health, parental involvement at its best is ensuring consistency in health promotion practices in the school and home environments [23, 24] and sharing responsibilities [24]. With the schools being fully or partially virtual, it has become more difficult to promote health collectively and achieve health-promoting objectives.

## Changing roles and responsibilities of school leaders and teaching and non-teaching staff

This global pandemic has brought school leaders unrelenting pressure, sleepless nights, and limited room to manoeuvre [25]. This pressure in an already demanding job has increased the likelihood of health problems, as well as potential negative effects on school quality and effectiveness. School leaders have a substantial role in school health promotion, especially in health-promoting change processes. In addition to traditional tasks, such as budget and staff management, the COVID-19 pandemic has also introduced the organisation of school closures and re-openings, all while ensuring compliance with public health guidelines. This has required intensive intersectoral cooperation and the ability to deal with health-related information (i.e. health literacy) to make informed decisions [26, 27].

During the pandemic, teachers have also found themselves overworked and stressed. This unexpected situation has often pushed them to reinvent themselves in multiple ways [28]. Faced with the need to learn how to use technology quickly [29] and to adapt their pedagogical models to remote learning environments, they have also had to look after their elderly relatives and children while living in a context of great uncertainty [30]. Yet, in the midst of this crisis, many educators have discovered their own coping strategies and the importance of developing their socioemotional skills.

Given the meaningful amount of time teachers and students spend together, teachers play a key role in the teaching of socioemotional skills [31], as well as skills related to daily health behaviours. Although many countries’ national curricula support the teaching of these skills, clear guidelines on how to implement them actively, especially in the context of emergency situations such as the pandemic, might be lacking. Adequate support and capacity building for teachers to address students’ learning losses are emphasised in the recommendations on schooling during COVID-19 [10]. Moreover, as teachers’ roles in the post-COVID-19 era continue to extend beyond the teaching of their assigned subjects [32], it is important to ensure that teachers are equipped to support children and youth in meeting their everyday needs and facing their problems [33].

Along with teachers, students encounter non-teaching staff ranging from administrators to cleaning staff each day. All these persons are important ‘building blocks’ in the lives of children and youth [34], by contributing to a socially nurturing atmosphere, acting as role models, and bringing their own professional contributions to daily school life. During the pandemic, many of these blocks and connections have been missing. For instance, as a central component of HPSs, school health services provided by health workers to students [35] might have encouraged the postponement or cancellation of appointments due to an increased need for professionals in other healthcare sectors.

## How can HPSs help to build back after the pandemic?

These perspectives bring out the multifaceted and rich, complex environment of schools during a global pandemic. Health has become a central issue for every school [7]. The HPS has been favoured as the most promising approach that goes beyond individual behaviour changes by addressing the physical and social environments and taking into account all groups involved in and with schools [8]. The HPSs can develop evidence-based approaches that prioritise children and youth’s health [36]. Related to COVID-19, the HPS approach has been shown to promote learning and physical, mental, and social health; address inequalities; and reinforce resources useful to increase health regulation adherence [37].

Despite the progress made in recent years, we now face the threat that the whole-school approach to health might be reduced to behavioural (e.g. hygiene-related) approaches. Moreover, related to the learning gap, schools in the future may tend to invest their time primarily in core subjects, while health – especially a broad understanding of health – receives less attention. However, it should be emphasised that the HPS is not only a resource for promoting individual health, but also an essentil input for teaching and learning processes, as well as school success and quality [38]. Health and education intertwine and require complex intervention strategies routed in a positive concept of health, addressing multiple topics and target groups.

Although access to the school building has been limited due to COVID-19 restrictions in many parts of the world, activities aimed at promoting an atmosphere of social support and connectedness have become essential during crisis [39, 40]. Following the recommendations for schooling during COVID-19 [8], school closures should be considered as the last measure to manage the pandemic. Longer closures are likely to contribute to widening educational and health inequities and, thus, should be avoided. If school closures exist, the continuity of substitute and adapted services, appropriate skills, and support should be offered, and special emphasis should be put on schools in deprived areas and for children who are at risk of dropping out of school or living in vulnerable situations. Regarding families, every effort should be made to engage parents in school activities to ensure that the links between home and school are maintained. The importance of reciprocal communication between home and school is even more significant in rapidly changing conditions, and policies to support parents are needed [41].

School leaders are in an important position for developing strategies and policies that promote social support and cohesion among all those involved in and with schools. Health-promoting leadership focuses on inspiring school staff and on developing a culture of health based on shared goals and values translated into policies [42]. However, in contrast to a formal understanding of leadership, the maintenance and promotion of health is the responsibility of many, not merely an individual alone. Creating capacity building opportunities across all levels within the school is necessary to enable the participation in activities which maintain and promote health. While the pandemic has significantly changed the role of teachers, it is critical to continue investing in their technical, psychosocial, and emotional support [41]. Further, the participation and visibility of non-teaching staff in school health promotion processes and structures calls for action. Not much is known about the health situation of non-teaching staff and on how the pandemic has affected their working and health situations. With this in mind, more research efforts should be undertaken in the future, and this is also in light of the fact that non-teaching staff is a key source of support for student health and educational success. School connectedness, in the sense of belonging to the school community, is growing in importance after ragged school semesters and needs to be rebuilt again. Fostering interaction through specific activities involving members of the entire school is helpful in building connectedness [43].

We emphasise a bottom-up equity and democratic approach by considering young people’s perspectives and participation in developing intervention programmes and structures to overcome the problems resulting from the COVID-19 crisis. Improving health literacy can help in tackling the pandemic, infodemic, and associated threats [11, 44]. More data are needed to be able to understand this period and its consequences for children’s academic progress, health and wellbeing, and the educational practices [3]. In preparing for the future, the use of evidence-based knowledge and learning from current experiences is essential. For sustainability and further development of school health promotion in this direction, we recommend collaborative and empowering activities that focus on developing relevant skills and transmitting values that reflect the holistic approach of HPSs.
